# GWAS implicated risk variants in different genes contribute additively to increase the risk of coronary artery disease (CAD) in the Pakistani subjects

**DOI:** 10.1186/s12944-018-0736-2

**Published:** 2018-04-19

**Authors:** Saleem Ullah Shahid, N. A. Shabana, Abdul Rehman, Steve Humphries

**Affiliations:** 1 Department of Microbiology and Molecular Genetics, University of the Punjab, Lahore, Pakistan; 2 Center for Cardiovascular Genetics, British Heart Foundation Laboratories, University College London, London, UK

**Keywords:** Coronary artery disease, Genetic risk score, Allele discrimination techniques

## Abstract

**Background:**

Coronary artery disease (CAD) remains the single most important cause of mortality worldwide. Many candidate and GWAS genetic variants have been identified in the recent years. In the current study, we selected six SNPs from various genes that have originally been identified in GWAS studies and examined the association of SNPs individually and as a genetic risk score (GRS) with CAD and blood lipid levels in the Pakistani subjects.

**Methods:**

Six hundred twenty-four (404 cases and 219 controls) subjects were genotyped for variants rs10757274 in CDKN2A gene, rs17465637 in MIA3 gene, rs7025486 in DAB2IP gene, rs17228212 in SMAD3 gene, rs981887 in MRAS gene and rs1746048 in CXCL12 gene, by TaqMan and KASPar allele discrimination techniques. Serum lipid parameters were measured using commercially available kits. Statistical analyses were done using SPSS version 22.

**Results:**

Individually, the single SNPs were not associated with CAD (*p* < 0.05). However, the combined GRS of 6 SNPs was significantly higher in cases than controls (4.89 ± 0.11 vs 4.58 ± 0.08, *p* = 0.024). Among blood lipids, GRS showed significant positive association with serum triglycerides levels (*p* = 0.022).

**Conclusion:**

The GRS was quantitatively associated with CAD risk and showed association with serum triglycerides levels, suggesting that the mechanism of these variants is likely to be in part at least through creating an atherogenic lipid profile in subjects carrying high numbers of risk alleles.

**Electronic supplementary material:**

The online version of this article (10.1186/s12944-018-0736-2) contains supplementary material, which is available to authorized users.

## Background

Coronary artery disease (CAD) is a multifactorial chronic disorder of coronary arteries progressing silently and usually has established to an advance stage by the time symptoms start appearing [1]. In CAD, a fatty material known as plaque deposits inside the coronary arteries which narrows their lumen and restricts flow of blood to myocardial muscles, the process is known as atherosclerosis. Although born with clear coronary arteries having wide lumen, the first streaks of fatty material may appear in the teenagers [2] but the disease is asymptomatic until the plaque has grown enough to compromise the flow of blood [3]. Pakistan is a developing South Asian country with a total population of 184.35 million in 2012–13 and is the 6th most populated country of the world [4]. The major part of the population (67.5%) lives in rural areas which has been estimated to bear the greatest burden of heart disease due to lack of awareness and unavailability of medical facilities [5]. The prevalence of CAD is even high in Pakistan [6] with over 30% of the population (> 45 years of age) being affected by the disease [7]. The disease burden has almost doubled in urban Karachi since 1970 [8]. Punjab is the largest province of Pakistan in terms of population accounting for more than 45% of the entire population [9]. However, there is a paucity of data on the estimates of risk factor of CAD or of its control status in Punjab, Pakistan [10]. According to latest WHO reports, cardiovascular diseases (CVD) are among the biggest non-communicable killers in Pakistan and CAD represents a major type of CVD (http://www.who.int/countries/pak/en/).

Both genetic and environmental factors contribute to the development of disease. These conventional risk factors (CRF) for the development of CAD can be classified into non-modifiable and modifiable categories. The non-modifiable risk factors are older age, male gender and having a family history of CAD. Modifiable CAD risk factors include hypertension, diabetes, blood lipid levels, obesity, smoking, unhealthy use of alcohol, sedentary life style and psychosocial stress [7, 11]. The non-modifiable factors are innate and cannot be changed whereas; modifiable risk factors can be changed through life style/behavioural changes or drug interventions.

The plasma biomarkers of lipid metabolism, thrombosis, inflammation and metabolic status are important components of CAD risk analysis. Blood lipid levels, coagulation profile, C-reactive proteins, fibrinogen and LP(a) levels were the biomarkers which had been in use for CAD risk stratification [12]. Genetic determinants of CAD risk analysis mostly included the genes regulating these biomarkers. Compared to candidate gene studies which target a gene known to have some role in cardiac physiology, GWAS simultaneously target hundreds of variants. Since this is not a hypothesis driven approach, it is considered to be more unbiased and has the potential to reveal new genetic associations with CAD [13, 14]. The CAD associated SNPs identified from GWAS are mostly intergenic and were unlikely to be explored by candidate gene studies. However, because the effect size associated with each variant is modest and generally common variants have low effect size, it leaves much of the CAD heritability unexplained [13].

In this study, we have included 6 GWAS SNPs and aimed to investigate the biochemical and genetic risk factors of CAD in the Pakistani people. The SNPs included (with nearest annotated gene) were rs10747275 (in cyclin dependent kinase inhibitor 2A gene, *CDKN2A*) [15], rs17465637 (in Melanoma inhibitory activity protein 3 gene, *MIA3*) [15], rs7025486 (in Disabled homolog 2-interacting protein gene, *DAB2IP*) [16], rs17228212 (in Mothers against decapentaplegic homolog 3 gene, *SMAD3*) [17], rs9818870 (in muscle RAS oncogene homolog gene, *MRAS*) [18] and rs1746048 (in C-X-C motif chemokine ligand 12 gene, *CXCL12*) [17]. These SNPs were selected for analysis in Pakistani subjects because 1) Pakistani population represents a unique ethnic group which allows the study of concentrated risk genetic markers 2) the selected SNPs have been reported to modulate serum lipids in Caucasians therefore their analysis in Pakistan can provide the information on the relationship of these genetic variants with lipids and 3) these SNPs have not been previously investigated in the Pakistani population and the current study is the first report of their study in our population. The biochemical risk factors included were lipid profile parameters namely total cholesterol (TC), very low density lipoprotein (VLDL), LDL-C, high density lipoprotein cholesterol (HDL-C) and triglyceride (TG) levels.

## Methods

The analyses were carried out in a case-control study design. The study was done at the Department of Microbiology and Molecular Genetics, University of the Punjab and Center for Cardiovascular Genetics, University College London. A total of 623 subjects (404 diagnosed CAD cases and 219 healthy controls) were recruited. The criteria for the selection of study subjects has been described in detail previously [19]. The CAD patients were selected from tertiary care hospitals of the province of Punjab, Pakistan. These were diagnosed cases based upon the ECG, cardiac echo, radiologic and troponin T/I levels. Only those CAD cases were selected which were recently diagnosed and had not started lipid lowering or antihypertensive drugs therapy. The controls were apparently healthy subjects, not having any history of an early CAD in their family. It was taken care that cases and controls represented all the socioeconomic groups. Subjects with obesity (BMI > 26Kg/m^2^ for Asian populations [20]) were excluded from the study to reduce the possible confouders. Any subject with infection was excluded from the study. All participants gave a written informed consent. The study was approved by the ethics committee, University of the Punjab, Lahore and all the procedures were in compliance with the Helsinki Declaration.

### Genotyping

The DNA was extracted from whole blood leucocytes using Wizard genomic DNA purification Kit (Promega, USA) and were quantified using nanodrop (ND-8000, USA). The genotyping was carried out in especially designed 384 well plates (Micro Amp). All the six SNPs were genotyped by KASPar allele discrimination technique according to the method given in section 2.22. For all the SNPs, homozygous and heterozygous genotypes were not properly separated after the standard KASPar cycle. Three extra cycles were given to the plates after which the genotypes were properly clustered. The genotypes were also randomly confirmed by direct DNA sequencing. After amplification, the results were analyzed on the ABI Prism 7900HT (Applied Biosystems/Life Technologies) and the genotypes were called using sequence detection software (SDS), version 2.0. The quality check of genotyping techniques was maintained by the inclusion of non-template controls (NTCs).

### Statistical analysis

The results were analyzed using statistical package for social sciences (SPSS) IBM, version 22. The continuous variables like blood lipid levels were compared between cases and controls using independent sample student *t* test. Hardy Weinberg equilibrium was accessed by a χ^2^ goodness of fit test. The categorical variables such as risk allele frequencies (RAFs) were compared between cases and controls using χ^2^ test. Since CAD is a binary variable, the association of the SNPs with CAD was examined using binary logistic regression. The effect of a range of risk alleles was calculated by GRS quartiles selecting the lowest through highest GRS levels. Blood lipid levels across different number of risk allele in GRS were calculated by one way analysis of variance (ANOVA) by selecting lipids as dependent variable and genescore as the factor affecting lipids. The effect size/β effect which is per risk allele effect of GRS on lipid levels (increase/decrease) was calculated by running a linear regression model (age and gender adjusted). As four SNPs were simultaneously analyzed, a corrected *p*-value (0.05/4 = 0.01) was used as a significance cutoff.

### Constructing a GRS

The GRS was calculated by adding the number of risk alleles at all the loci included in the study. The risk alleles were assumed to be acting in additive manner, i.e., each risk allele had an equal contribution to the outcome and each risk allele was coded as 1. So the protective homozygous genotype with no risk allele was coded 0, the heterozygous individual carrying one risk and one normal allele was coded as 1 and the risk homozygous individual having both risk alleles was coded as 2. In this way the GRS of an individual can range from 0 (no risk allele) to 12 (with all the alleles being risk alleles for 6 loci).

## Results

The basic features of the SNPs, including chromosomal location and the nearest annotated gene are presented in Additional file 1: Table S1. The call rate of all the SNPs was > 95% and all were in Hardy Weinberg equilibrium collectively as well as in cases and controls separately (*p* > 0.05). The selected SNPs were not in linkage disequilibrium as tested by the pairwise LD calculator available at http://archive.broadinstitute.org/mpg/snap/ldsearchpw.php. The study power assuming a standard deviation of 1 and with a sample size of 623 was 81%.

The risk allele frequencies in cases and controls are given in Table 1. The RAFs were higher in cases compared to controls, but the difference was not statistically significant (*p* > 0.05). The number and percentage of individual genotypes (common homozygotes, heterozygotes and risk homozygotes) were calculated. The percentage of the risk homozygotes was higher in cases than controls (Table 2). The per allele CAD odds ratios of SNPs with confidence intervals and significance levels is shown in Table 3. None of the SNPs was found to be significantly associated with CAD in the studied samples.

**Table 1:** Comparison of risk allele frequencies between cases and controls

SNP			RAFs			
	Alleles	Risk allele	Cases	Controls	*p	Total
rs10757274	A/G	G	0.505	0.463	0.162	0.490
rs17465637	C/A	C	0.651	0.621	0.292	0.640
rs7025486	G/A	A	0.318	0.315	0.913	0.317
rs17228212	T/C	C	0.207	0.176	0.190	0.196
rs9818870	C/T	T	0.094	0.087	0.670	0.091
rs1746048	C/T	C	0.675	0.630	0.114	0.659

RAF is risk allele frequencies *p is chi square *p*-value

**Table 2:** The genotypes of the SNPs

SNP/alleles	CAD (404)	Control (219)
rs10757274		
AA	107 (26.5%)	60 (27.4%)
AG	186 (46.0%)	115(52.5%)
GG	111 (27.5%)	44 (20%)
rs17465637		
AA	46 (11.4%)	31 (14.2%)
AC	190 (47%)	104 (47.5%)
CC	168 (41.6)	84 (38.4%)
rs7025486		
GG	190 (47%)	99 (45.2%)
AG	171 (42.3%)	102 (46.6%)
AA	43 (10.6)	18 (8.2%)
rs17228212		
TT	252 (62.4%)	151(68.9%)
CT	137 (33.9%)	59 (26.9%)
CC	15 (3.7%)	9 (4.1%)
rs9818870		
CC	333 (82.4%)	184 (84%)
CT	66 (16.3%)	32 (14.6%)
TT	5 (1.2%)	3 (1.4%)
rs1746048		
TT	40 (9.9%)	33 (15%)
CT	183 (45.3%)	96 (43.8%)
CC	181 (44.8)	90 (41.1%)

**Table 3:** Per allele CAD odds ratios of the studied SNPs

SNP	OR	95% C.I	Significance
rs10757274	1.18	0.93–1.5	*p* = 0.17
rs17465637	1.14	0.89–1.5	*p* = 0.29
rs7025486	1.01	0.79–1.3	*p* = 0.91
rs17228212	1.22	0.91–1.65	*p* = 0.19
rs9818870	1.09	0.73–1.62	*p* = 0.68
rs1746048	1.22	0.95–1.6	*p* = 0.12

OR is odds ratio, C.I is confidence interval

Whether there was an association between any of the SNPs and lipid traits, including LDL-C, HDL-C, TC and TG levels was also determined. As expected for these GWAS SNPs, no significant association of any SNP was observed with any of the lipid traits examined. For all the SNPs, per allele beta estimates for association with lipid traits were very low and statistically non-significant. The only significant associations which were observed were that of the SNP *MIA3* rs17465637 with TG and *CXCL12* rs1746048 with TC levels. The beta effect of each risk allele of *MIA3* rs17465637 for TG was 10.2 ± 4.1 mg/dl (*p* = 0.01) and the beta effect of each risk allele of *CXCL12* rs1746048 for TC was 6.5 ± 3.1 mg/dl (*p* = 0.04). The association of rs1746048 with TC, however, vanished at a corrected *p*-value (0.01) (Table 4).

**Table 4:** Association of the SNPs with lipid traits individually

SNP	Genotype	LDL-C	HDL-C	TC	TG
rs10757274	AA	99.6 ± 28.2	53.4 ± 19	196.7 ± 52.3	205.4 ± 68.9
	AG	97.3 ± 27	53.9 ± 17.4	195.2 ± 51.8	203.2 ± 71
	GG	99.6 ± 27.1	50.8 ± 14.6	197.6 ± 54	203.4 ± 68.2
	β(Se)	0.06 (1.52)	1.27 (0.96)	0.5 (2.9)	1.01 (3.9)
	*p*-value	0.97	0.188	0.88	0.794
rs17465637	AA	96.3 ± 23	54.8 ± 18.1	190.7 ± 52.3	196.5 ± 68.8
	AC	99.4 ± 26.8	53.6 ± 16.4	196.9 ± 52.0	206.6 ± 68.2
	CC	98 ± 29.1	51.8 ± 17.9	197.1 ± 53	217.0 ± 76
	β (se)	0.17 (1.6)	1.6 (1.0)	2.3 (3.1)	10.2 (4.1)
	*p*-value	0.92	0.13	0.46	0.01
rs7025486	GG	98.3 ± 26.5	53.7 ± 17.0	195.4 ± 50.7	203 ± 70.7
	AG	99.0 ± 28.0	52.9 ± 17.7	197.9 ± 54.1	206.7 ± 70.4
	AA	96.8 ± 28.5	50.0 ± 16.0	192.5 ± 53.4	194.4 ± 61
	β (Se)	0.19 (1.7)	1.5 (1.0)	0.07 (3.2)	1.3 (4.3)
	*p*-value	0.91	0.16	0.98	0.76
rs17228212	TT	98.3 ± 25.5	53.1 ± 17.2	195.4 ± 52.8	204.5 ± 68.4
	CT	99.2 ± 30.2	52.7 ± 17.8	198.3 ± 52.2	203.3 ± 71
	CC	95.8 ± 33.3	53.1 ± 14.6	192.5 ± 50	195.6 ± 81
	β (Se)	0.07 (2)	0.3 (1.2)	1.2 (3.7)	2.5 (5)
	*p-*value	0.97	0.83	0.76	0.62
rs9818870	CC	97.7 ± 26.6	53.3 ± 17	196.8 ± 53.8	203.2 ± 68
	CT	102.4 ± 29.6	52 ± 18.2	192.4 ± 43.7	203.3 ± 76.5
	TT	102.9 ± 41.8	48.3 ± 17.7	206.6 ± 63.8	252.5 ± 74.0
	β (Se)	4.1 (2.6)	1.6 (1.6)	1.9 (5)	6.7 (6.7)
	*p*-value	0.11	0.35	0.7	0.32
rs1746048	TT	97.1 ± 28.2	53.5 ± 19.2	185.8 ± 50.3	205.1 ± 61.2
	CT	100.4 ± 26.6	52.6 ± 16.9	195.1 ± 51	205.9 ± 72.9
	CC	96.9 ± 27.8	53.3 ± 17.1	200.1 ± 54.1	201.3 ± 68.6
	β (Se)	1.2 (1.6)	0.1 (1)	6.5 (3.1)	2.7 (4.2)
	*p*-value	0.47	0.9	0.04	0.51

β (Se) is the effect size with standard error. Values are mentioned as mean ± SD. The analyses were adjusted for age and gender

The genetic risk score constructed by adding the risk allele count for each study subject was analyzed in relation to the CAD status. The mean GRS in cases was higher in the cases than in the controls with a marginal significance (4.89 ± 0.11 vs 4.58 ± 0.08, *p* = 0.024). The bivariate regression analysis with CAD as a dependent and GRS as an independent variable revealed a significant association of GRS with CAD with an Odds ratio of 1.12 (CI: 1.01–1.24, *p* = 0.022). We divided the GRS into quartiles and observed the highest frequency of the subjects in the interquartile range. The mean values for lipid parameters were checked across the quartiles and an increase in the serum TG levels was observed in the top quartile (*p* = 0.022). The β-effect to check the per risk allele increase in the lipid parameters was quantified using linear regression across the quartiles. The results of these analyses are summarized in the Table 5.

**Table 5:** Comparison of Mean lipid parameter values across GRS quartiles

Parameter	Q1	Q2	Q3	β-effect	*p*-value
TC (mg/dL)	192.6 ± 4.3	197.2 ± 2.4	196.6 ± 3.3	0.049	0.676
TG (mg/dL)	160.2 ± 6.1	202.4 ± 3.2	212.4 ± 6.1	0.064	0.022
HDL-C (mg/dL)	53.8 ± 1.5	53.1 ± 1.8	48.3 ± 3.1	−0.064	0.536
LDL-C (mg/dL)	93.9 ± 2.7	93.6 ± 1.5	104.8 ± 1.3	0.020	0.449

Q1: GRS 0–4, Q2: GRS 5–8, Q3: GRS 9–12

## Discussion

In this study, we have replicated the association of 6 SNPs with CAD in a group of Pakistani people. The studied SNPs were GWAS hits for CAD in people of European ancestry. Out of these 6 SNPs, 4 (rs10757274, rs17465637, rs9818870 and rs1746048) were also confirmed in the recent CARDIoGRAMPlusC4D consortium study [21]. All of these SNPs except *DAB2IP* rs7025486 were initial hits of Welcome trust case control (WTCCC) and German MI studies [17].

In the current study, we could not find a statistically significant difference in the between the CAD cases and controls, therefore these SNPs cannot be considered as independent risk factors of CAD in the studied population. This might be a power issue as the GWAS SNPs have been studied with thousands of samples. The ethnicity may also be another factor as the LD patterns differ in different ethnicities and genetic factors also interact with that of the environmental factors.

In *CDKN2A* rs10757274, the proportion of heterozygotes was higher in controls than cases (52.5% vs. 46.0%) but the risk homozygotes were higher in cases than controls (27.5% vs.20%). Whereas, this SNP is the most frequently and consistently associated GWAS hit for CAD. The SNP was strongly associated with CAD in a cohort of Asian Indians [22]. The SNP also exhibited association with T2DM which is a CAD risk factor [23]. The region *9p21* is overlapped by *ANRIL* which is a non-coding RNA gene [24]. The elevated expression of *ANRIL* is reported in vascular tissues, smooth tissues and monocytes and all have role in atherogenesis [25]. The *ANRIL* gene is considered to modulate the expression of neighbouring genes such as *CDKN2A* and *CDKN2B*. Its role is also reported in plaque stability, thrombogenesis and vascular endothelium remodelling [26]. The variants at the region *9p21* were associated with increased expression of the *ANRIL* gene which in turn is proatherogenic [27]. So far the *CDKN2A* rs10757274 has been reported to be associated with CAD in many diverse ethnicities but it failed to improve net reclassification index or discrimination of CAD when considered along with other conventional CAD risk factors [28].

The risk allele of *MIA3* rs1746537 was not associated with CAD but was increasing TG levels in our study. The association with blood lipids had not been confirmed in a previous study on about 25,000 individuals [29]. So this SNP might be causing CAD by raising TG levels in Pakistani population or it may be a chance finding or false positivity due to multiple testing. The association of this SNP with CAD has been reported in American Caucasians [30]. Whereas the association remained inconsistent in three follow up studies on Chinese people, however, a subsequent meta-analysis on five Asian populations confirmed the association with CAD in this ethnic group [31]. The association with CAD without affecting plasma lipids and other CAD biomarkers was also found in Japanese population [32]. The SNP *MIA3* rs17465637 was identified as a possible CAD risk after combining the results from WTCCC and German MI family study. The association was reported to be about 8% which was the second strongest association [17].

No association of *DAB2IP* rs7025486 with CAD or any of the lipid traits was observed in our samples whereas; a strong association of this SNP with CAD was reported in a previous meta-analysis [16]. Loss of DAB2 interacting protein due to gene methylation increases cell proliferation and decreases apoptosis in certain type of cancers. The protein normally reduces cell proliferation and accelerates cell senescence and its direct role in cardiac functioning has not been shown yet [33]. The SNP *SMAD3* rs17228212, as expected was not associated to any of the lipid parameters studied. However, the SNP has been reported to be associated with risk of cardiovascular disease and a subclinical atherosclerosis [34]. The SNP *MRAS* rs9818870 was also not found to be associated with CAD and lipid parameters. However, the SNP has been proved to be a predictor of cardiovascular disease risk in CAD free individuals [35]. The minor allele frequency of this SNP in Caucasians was significantly higher than Chinese according to HAPMAP II [36]. The SNP *CXCL12* rs1746048 was not associated with CAD; however the minor allele was raising TG levels. This may be a new finding observed in Pakistani people and need to be replicated in this ethnic region. However, a strong association of this SNP with CAD was observed in a large meta-analysis and case control study on Han Chinese population [37]. Similarly, the SNP was also associated with MI in an additive manner in Chinese people [38].

In this study, the GRS was calculated assuming that all the SNPs had an equal effect on the outcome and act additively. However, this may not always be the case because the effect size of some SNPs is relatively high while some have more modest effects. At the same time, some SNPs may be positively correlated while others can be negatively correlated with the outcome; we therefore adjusted coding for all the SNPs such that they had same direction of association with CAD. The GRS was also significantly associated with CAD risk. However, the effect sizes of the SNPs may vary among ethnicities as linkage disequilibrium and allele frequencies vary [39].

The study had the limitations of small sample size, inability to include more biochemical parameters and inclusion of only a certain number of SNPs. As none of the SNPs showed significant association with the CAD individually, these genetics markers could not independently explain risk susceptibility to CAD. Thus, the genotype has to be considered in relation to the phenotype and clinical presentation due to the multifactorial nature of CAD.

## Conclusion

In conclusion, the studied samples were although within Hardy Weinberg equilibrium, but a significant association of risk alleles with CAD could not be proved, although RAFs were always higher in CAD cases than controls. However, the study replicated the findings that these GWAS hits were not associated to the blood lipid traits as in previous GWAS studies. It is thus useful to use a panel of risk variants that can be used to construct a GRS which provides advantages over the use of single SNPs.

## Additional file

Additional file 1: **Table S1.** Basic feature of the SNPs. (DOCX 14 kb)
