# Supplementary material for: GWAS implicated risk variants in different genes contribute additively to increase the risk of coronary artery disease (CAD) in the Pakistani subjects
Source: Lipids Health Dis. 2018 Apr 19;17:89. doi: 10.1186/s12944-018-0736-2 (PMC5909255; doi:10.1186/s12944-018-0736-2)
Supplement: Supplementary file 1 — Table S1. Basic feature of the SNPs. (DOCX 14 kb) [file 12944_2018_736_MOESM1_ESM.docx]

**Additional file 1: Table S1. Basic feature of the SNPs**

| Gene | CHR | SNP | Call rate % | HWE-*p* | | |
| --- | --- | --- | --- | --- | --- | --- |
|  |  |  |  | Cases | Controls | Total |
| *CDKN2A* | 9P21,3 | rs10757274 | 96 | 0.111 | 0.408 | 0.405 |
| *MIA3* | 1q41 | rs17465637 | 97 | 0.482 | 0.896 | 0.538 |
| *DAB2IP* | 9q33,2 | rs7025486 | 98 | 0.626 | 0.242 | 0.766 |
| *SMAD3* | 15q22,33 | rs17228212 | 96 | 0.493 | 0.298 | 0.978 |
| *MRAS* | 3q22,3 | rs9818870 | 96 | 0.405 | 0.249 | 0.180 |
| *CXCL12* | 10q11,21 | Rs1746048 | 96 | 0.525 | 0.378 | 0.926 |

Call rate is the percentage of the samples which were amplified during genotyping that SNP.
